# Heterogeneity of macrophage infiltration and therapeutic response in lung carcinoma revealed by 3D organ imaging

**DOI:** 10.1038/ncomms14293

**Published:** 2017-02-08

**Authors:** Michael F. Cuccarese, J. Matthew Dubach, Christina Pfirschke, Camilla Engblom, Christopher Garris, Miles A. Miller, Mikael J. Pittet, Ralph Weissleder

**Affiliations:** 1 Center for Systems Biology, Massachusetts General Hospital, 185 Cambridge Street, CPZN 5206, Boston, Massachusetts 02114, USA; 2 Department of Radiology, Massachusetts General Hospital, 185 Cambridge Street, CPZN 5206, Boston, Massachusetts 02114, USA; 3 Department of Systems Biology, Harvard Medical School, 200 Longwood Avenue, Boston, Massachusetts 02115, USA

## Abstract

Involvement of the immune system in tumour progression is at the forefront of cancer research. Analysis of the tumour immune microenvironment has yielded a wealth of information on tumour biology, and alterations in some immune subtypes, such as tumour-associated macrophages (TAM), can be strong prognostic indicators. Here, we use optical tissue clearing and a TAM-targeting injectable fluorescent nanoparticle (NP) to examine three-dimensional TAM composition, tumour-to-tumour heterogeneity, response to colony-stimulating factor 1 receptor (CSF-1R) blockade and nanoparticle-based drug delivery in murine pulmonary carcinoma. The method allows for rapid tumour volume assessment and spatial information on TAM infiltration at the cellular level in entire lungs. This method reveals that TAM density was heterogeneous across tumours in the same animal, overall TAM density is different among separate pulmonary tumour models, nanotherapeutic drug delivery correlated with TAM heterogeneity, and successful response to CSF-1R blockade is characterized by enhanced TAM penetration throughout and within tumours.

## Abstract (web summary)

Tumour-associated macrophages (TAM) can be used as prognostic indicators in cancer. Here, the authors establish a platform for high-throughput 3D microscopy in murine lung carcinoma that allows to visualize TAMs infiltration throughout the entire lung, response to CSF-1R blockade and nanoparticle drug delivery.

Tumour microenvironments often include vast numbers of seemingly normal host cells, including a diverse immune cell population, which can potently regulate cancer progression^1^,^2^. Among immune cells, tumour-associated macrophages (TAM) have recently attracted much attention as they play key roles in tumour spread and response to therapy: TAM can not only accelerate the progression of untreated tumours^3^,^4^,^5^ but also markedly influence the efficacy of anticancer drugs^6^,^7^,^8^,^9^. Furthermore, targeting TAM themselves, for instance via colony-stimulating factor 1 receptor (CSF-1R), can control the progression of some murine^1^ and human^10^ tumours. However, most of our knowledge on TAM comes from histological examinations and *in vitro* profiling^7^,^11^,^12^,^13^, whereas there remains a significant knowledge gap on how TAM function *in vivo*. Moreover, tumour biology can vary between the surface and the centre of a single nodule with regard to proliferating and quiescent cells that may affect TAM distribution^14^. Other microenvironmental features such as hypoxia and intratumoral lactic acid can profoundly affect TAM polarization^15^. Information on the spatial distribution of TAM, their heterogeneity amongst different tumours or variability across a population of animals, however, is limited.

Imaging represents a unique approach to visualize tumours in three-dimensional (3D) and is typically done at two extremes: (i) the whole body level, covering entire organs, yet with the inherent limitation that minimum detectable tumour size is typically >1 mm^3^ (and often >125 mm^3^)^16^ and has not been useful to interrogate tumour microenvironment composition; and (ii) the microscopic level where individual cell types, such as host and tumour cells can be simultaneously identified^17^,^18^ but coverage is limited to a few mm^2^ and a depth of ∼100 μm before resolution is degraded by light scattering^19^. Naturally, there has been interest in mesoscopic scale imaging that allows survey of entire organs with the ability to zoom in onto individual groups of cells. The introduction of faster image acquisition^20^ and tissue clearing methods^21^,^22^,^23^,^24^ has drastically improved deep tissue imaging, primarily for neurobiology. Techniques for multichannel light microscopy with tissue-clearing approaches are rapidly evolving but some limitations remain for labelling and imaging tumours, such as slow antibody penetration (on the order of weeks for whole organs), uneven labelling, and cost^25^.

Driven by the biological question of how heterogenous TAM recruitment is to lung adenocarcinoma, we set out to develop a rapid whole-lung imaging protocol that reveals details on macrophage infiltration throughout the entire intact mouse lung. Furthermore, our approach maintains the ability to image cellular detail at desired locations throughout all lobes. Using preserved fluorescent protein, *ex vivo* molecular dyes, and intravenously delivered labels, we were able perform whole-organ tumour burden, host-cell analysis and drug-delivery assessment within days. We were specifically interested in addressing the following questions: (i) can tissue clearing and fluorescence microscopy be used to measure tumour burden with sufficient sensitivity; (ii) what is the heterogeneity of TAM infiltration across metastatic lung tumours^1^; and (iii) what is the effect of PLX3397, a competitive ATP inhibitor with potent specificity for CSF-1R and cKIT receptor tyrosine kinases on macrophage density, cellular distribution and ultimate tumour progression; and (iv) can one measure nanotherapeutic delivery to individual tumour nodules? We discovered that TAM infiltration is highly variable within and amongst lung tumours and does not decrease with successful PLX3397 treatment. Rather, successful therapy is characterized by spatial reorganization of overall TAM distribution. Thus, these findings open new ways of studying tumour and host-cell heterogeneity in whole organs.

## Results

### Tissue clearing for whole lung cellular imaging

**Figure 1:**
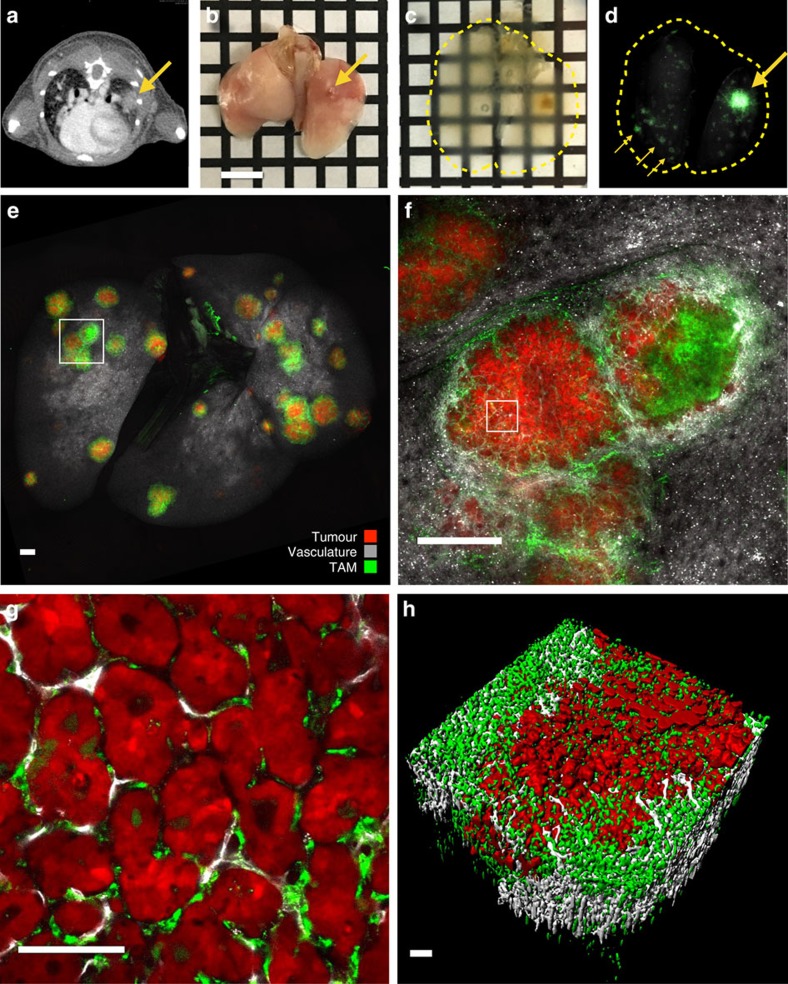
Clearing of lung tissue allows visualization of tumour burden and other biologically relevant features. (**a**) CT scan of KP-tumour-bearing mouse and identification of large lung tumour (big arrow). (**b**–**d**) Process of clearing and imaging lungs and identification of small tumours (small arrows). (**e**) Wide-field image of whole lung from KP tumour-bearing animal. (**f**) × 4 slice of a tumour from box in **e**. (**g**) × 20-magnification imaging slice of a single tumour from box in **f**. (**h**) Computational 3D-rendering of a whole-lung tumour nodule. Scale bars, 5 mm (**b**); 1,000 μm (**e**,**f**); 100 μm (**g**,**h**).

While computed tomography (CT) offers noninvasive detection of tumour nodules in the lung of live animals (Fig. 1a), resolution limitations typically prevent accurate analysis of total tumour burden in the mouse. Drawing insight from optical clearing methods currently being used in brain imaging, we extended their application to pulmonary imaging. To accomplish this, we derived a clearing method from the CUBIC protocol^26^, substituting whole-animal perfusion for a right-ventricular perfusion and use of a shorter post-perfusion fixation time (Table 1). We also identified that samples can be imaged in CUBIC 1 in the lung at similar fidelity as with the index-matched CUBIC-2 solution (Supplementary Fig. 7). Importantly, this modified protocol applies intravenous administration of imaging probes to stain cell and tissue compartments of interest with high fidelity. For example, pre-injection of fluorophore-tagged lectin and macrophage-targeting NPs enabled visualization of vasculature and TAM, respectively, throughout the organ. Labelling TAM by pre-injection was superior to post-clearing antibody labelling because it removed the time consuming blocking and staining steps of antibody labelling. Penetration of antibodies in cleared or permeabilized tissue can be slow, requiring more than 7 days in the brain and likely longer in dense tumour tissue (a tumour contains 5–10 × more cells per mm^3^ than healthy brain tissue^27^,^28^).

To image pulmonary carcinoma, we applied the clearing and imaging technique in a lung adenocarcinoma model, in which tumour cells from a KRAS and p53 mutant mouse (KP) were injected intravenously^29^,^30^,^31^. This model of metastatic disease differs from primary lung cancers which develop in aged KP mice^29^. The clearing method revealed many more and smaller KP lung tumour nodules than found by conventional micro-CT (μCT) (Fig. 1b–d). Tumour cells and nodules were easily identified by fluorescence imaging upon staining with nuclear markers, such as DAPI or SYTO dyes, as tumours exhibited higher cellular density relative to healthy lung tissue (Supplementary Fig. 1d). In a comparison between DAPI and GFP-expressing KP tumours, we observed a Spearman's rank correlation value of 0.95, indicating that both labelling procedures can be interchangeably used for tumour identification and segmentation. Other endogenous features could be imaged in cleared lungs, such as the biotin-rich airways with fluorophore-conjugated streptavidin^32^ (Supplementary Fig. 1e and Supplementary Movie 2) and collagen fibres with two-photon second-harmonic generation (Supplementary Fig. 1f). It is advantageous to use fluorochromes that bleach minimally and retain brightness through fixation and clearing. Several BODIPY, Alexa Fluor and cyanine analogues achieve this. Fluorescent proteins are sensitive to over-fixation and are only compatible with aqueous clearing methods (for example, sca/e, CUBIC and CLARITY).

After clearing, lung tissue was mounted in 2-mm-thick chambers (Supplementary Fig. 1a) and imaged using confocal microscopy, thereby rendering lungs transparent (Supplementary Fig. 10) and enabling complete observation of lung tumour burden at cellular resolution throughout the whole-tumour mass (Fig. 1e–g). Surveying the whole lung for measurement of tumour volumes and immune infiltrate was possible after reconstruction of a grid of × 2 image stacks (Fig. 1e). Finer detail on TAM localization and its relation to the tumour mass could then be ascertained at × 4 magnification (Fig. 1f), and cellular resolution could be obtained at × 10–20 magnification (Fig. 1g, × 20). Computationally rendering image stacks allowed visualization of tumours in three dimensions (Fig. 1h and Supplementary Movie 1, 1.7 mm^3^ tumour, × 10) so as to contextualize tumour, TAM and vascular architecture. We found that 2–6-mm-thick sample chambers were advantageous for high-resolution spot imaging, since a thin chamber allows the lobes to spread apart such that and any tumour in the chamber can be imaged with minimal distortion, scattering or absorption penalties through thicker tissues. Direct comparison of unrestricted and 2 mm chambers yielded similar results (Supplementary Fig. 9). While advantageous for moldable organs such as lung, intestine and pancreas, it would be more suitable to image slices of organs with more defined structure (kidney, brain and liver). Table 2 summarizes the different experimental steps and acquisition times required for pulmonary imaging at different resolutions. Finally, while it was ideal to image lungs immediately after clearing, they were also stored at 4 °C for several weeks with little loss of fidelity in imaging. Collectively, these data show that entire lungs can be surveyed and tumours imaged at cellular resolution within reasonable amounts of time.

### Quantitative analysis of immune infiltration

**Figure 2:**
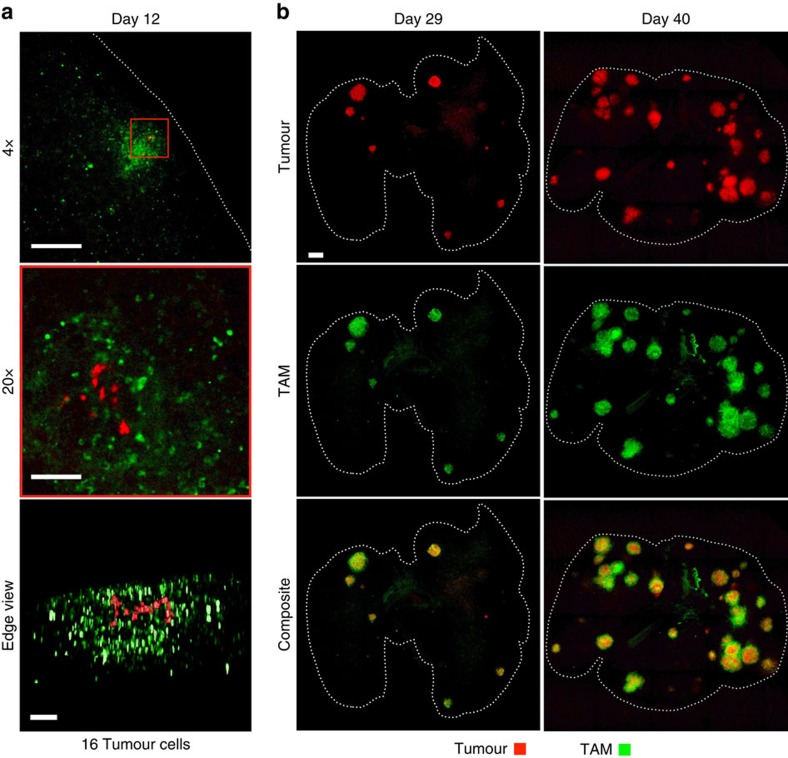
Detection and analysis of whole lung tumour burden. (**a**) Identification of TAM presence surrounding early-stage tumours. TAM infiltration is observed in response to 16 individual tumour cells within a single nascent nodule. Scale bars, 1,000 μm (top); 100 μm (middle/bottom). (**b**) Analysis of tumours in mice at two stages of disease progression revealing facile detection of tumours and a heterogeneous distribution of TAM and tumour location. Dashed lines outline the lung. Scale bars, 1,000 μm. For all, days denote time post inoculation.

Analysis of TAM content in the tumour stroma can yield strong prognostic^13^,^33^ and possibly therapeutic^9^ information. With this in mind, we sought to use the clearing method to visualize and quantify TAM invasion in individual tumours in whole lung tissue. Using GFP-labelled KP lung tumours and dextran NPs with avidity towards macrophage^34^, we found TAM to be associated with all tumour nodules investigated, at multiple stages of tumour progression. Figure 2 shows representative images of lungs cleared at 29 and 40 days after IV administration of 2.5 × 10^6^ KP tumour cells. Interestingly, TAM could be observed at even the earliest stage of tumour formation (day 12) when only a few cancer cells were present (Fig. 2a), and substantial heterogeneity was evident in tumour size, anatomical positioning and immune infiltration at later time-points (Fig. 2b).

**Figure 3:**
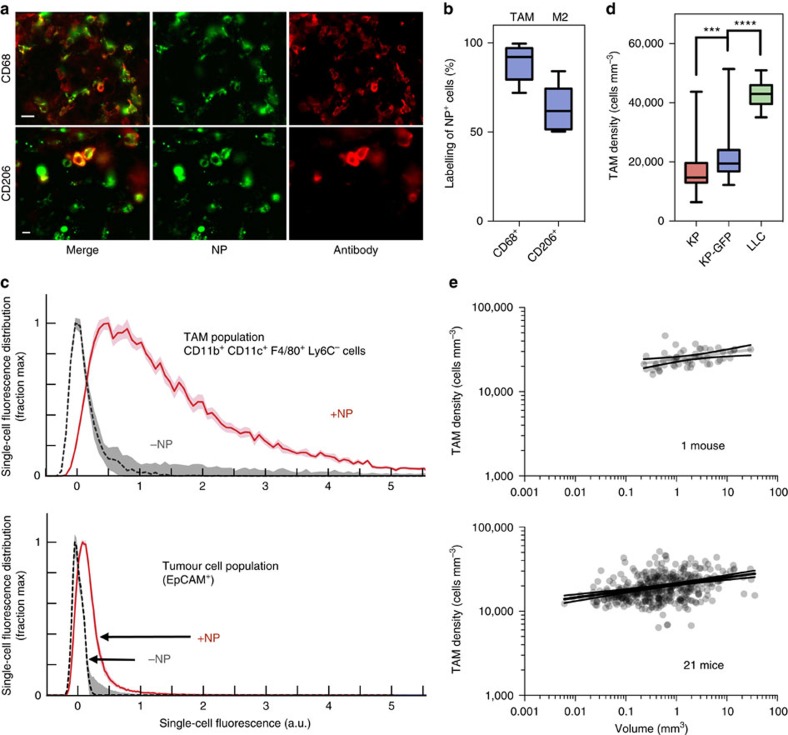
Identification and quantification of TAM infiltration. (**a**) IHC on formalin-fixed KP tumour sections, staining for CD68 and CD206 macrophage markers. Scale bar, 10 μm. (**b**) Analysis of NP^+^ cells by IHC for CD68 and CD206 (*n*=3 images). (**c**) NP was found in TAM (CD11b^+^ CD11c^+^ F4/80^+^ Ly6C^−^), whereas the NP did not accumulate in tumour cells (RFP). (**d**) Average TAM density in KP, KP-GFP and LLC tumour models as calculated from imaging (KP, *n*=158; KP-GFP, *n*=38; LLC, *n*=6; ***P=0.0003; ****P<.0001; one-way ANOVA). (**e**) Tumours plotted by size and TAM infiltrate for one mouse (top) and in relation to 20 other mice (bottom) to show range of tumour size and TAM infiltration. Scale bars, 10 μm. ANOVA, analysis of variance.

Building on previous reports of macrophage-selective uptake of dextran-coated iron oxide NPs^35^,^36^, we performed histology (Fig. 3a,b), flow cytometry (Fig. 3c) and imaging subcutaneously implanted HT1080 tumours in a NOD-SCID MERTK^GFP/+^ fluorescent reporter mouse (Supplementary Fig. 2) model to verify that the macrophage-avid NPs accurately identified TAM upon i.v. injection. Immunohistochemistry (IHC) showed that the majority of CD68^+^ TAM had internalized fluorescent NPs (at a level detectable by IHC), and about half were alternatively activated M2-like macrophages (Fig. 3a,b). Neutrophils are also phagocytic myeloid cells that can take up NP^35^. In the KP flank tumour model 72±3% (*n*=12) of the NP^+^ signal is due to CD11b^+^ CD11c^+^ F4/80^+^ TAM, and the remainder due to other phagocytes. Flow cytometry indicated that NP-positive cells were predominantly CD11b^+^ CD11c^+^ F4/80^+^ Ly6C^−^ and thus resembled TAM phenotypically (Fig. 3b). In cleared tumours grown in *MERTK*^*GFP/+*^ reporter mice, good co-localization was observed between the NP and GFP at × 10 magnification (*R*=0.89, Supplementary Fig. 2a). Computational single-cell segmentation was use for co-localization analysis of higher magnification images, and showed that 79% of GFP^+^ cells contained NP and 95% of NP-positive cells expressed GFP (Supplementary Fig. 2b). Thus, NP injection provides a convenient method to accurately identify TAM, and may represent a method for therapeutic drug delivery. For high-throughput quantification of cellular TAM levels, wide-field integrated fluorescence density of a series of tumours was computationally mapped to high-resolution images of the same series of tumours for automated cell segmentation and counting (Supplementary Fig. 3). A conversion factor between wide-field images and cell segmentation results was generated with a correlation coefficient of 0.9, *P*<0.0001 (two-tailed Student's *t*-test). Using this relationship, fluorescence density in other wide-field images was then used to infer cellular TAM density.

With a method to visually identify and measure tumour cells and TAM, we next sought to apply the analysis over many tumours and animals to obtain a more complete understanding of TAM heterogeneity in lung adenocarcinoma. The number and volume of pulmonary tumours in a given mouse largely depended on the time elapsed since tumour inoculation. Four weeks post inoculation (day 29), the average number of pulmonary metastases was 44 and ranged from 29 to 57. To expand the analyses, we investigated several hundred tumour nodules in 21 different mice (Fig. 3e). For example, in a given KP mouse, the average TAM density was 17,000 TAM mm^−3^ within a tumour, but this varied sevenfold among tumours of a similar size. In other words, across 1 mm^3^ tumours, TAM density varied from 8,000 to 60,000 in other similarly sized nodules. No discernible pattern was found in anatomic location or vascular features that predicted TAM density. We extended this analysis to a Lewis lung carcinoma model^37^ and GFP-expressing KP model. In both of these models, the TAM density in tumours was slightly higher (Lewis lung cell carcinoma (LLC): 43,000 TAM mm^−3^ tumour *P*<0.0001; KP-GFP: 21,000 TAM mm^−3^ tumour *P*=0.0001; one-way analysis of variance) than in the non-GFP KP model (Fig. 3d). In sum, these data show the variability of host-cell infiltration across pulmonary tumours within the same animal and across animals. Notably, no single lesion was devoid of innate immune cells.

### PLX3397 reduces tumour burden but not TAM density

The innate immune composition of the tumour microenvironment has emerged as an attractive therapeutic target and efforts are underway to modulate TAM numbers or molecular phenotypes to control cancer progression^9^. The presence of TAM can be essential for the efficacy of nanotherapeutics^31^,^35^ and the heterogeneity of TAM infiltrates among tumours may play a key role in varying response to therapy^38^. With respect to the latter, colony-stimulating factor receptor (CSF-1R) inhibitors have emerged as an attractive modulation method. Experimental CSF-1R blockade has been shown to result in altered TAM recruitment or M1/M2 polarization and lower tumour burden^1^,^39^. Some CSF-1R inhibitors are now progressing in clinical trials, which led us to investigate the effect of the CSF-1R and cKit inhibitor PLX3397 (refs 10, 40, 41) in the KP model. KP1.9 cells lack CSF-1R (Supplementary Fig. 8). Furthermore, PLX3397 was not toxic to KP1.9 cells nor RAW 264.7 cells at concentrations tested. To assess PLX3397 effects on TAM *in vivo*, tumours were grown for 21 days and mice were then treated with 30 mg kg^−1^ PLX3397 i.p. for 7 days. Lungs were then prepared for imaging.

**Figure 4:**
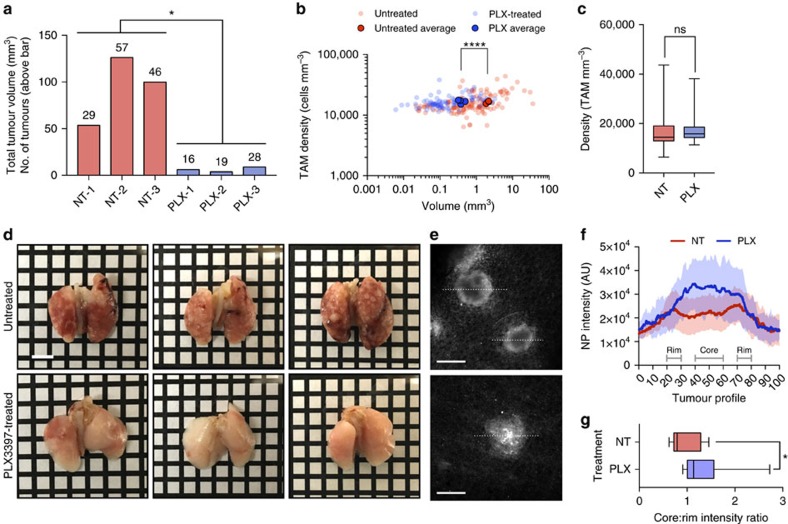
PLX3397 monotherapy decreases tumour burden and alters TAM density and infiltration. (**a**) Reduced tumour burden in PLX3397 (PLX) treated mice relative to untreated (NT) (*n*=6, *P*=.015; unpaired *t*-test). Bars represent total tumour volume with number of identified tumours indicated above each bar. (**b**) Plot of TAM density against tumour volume, showing PLX3397-treatment resulted in significantly smaller tumour nodules (*n*=423, *P*<.0001; unpaired *t*-test) (**c**) Data from **b** arranged to compare TAM densities in tumours from untreated and PLX3397-treated mice. (**d**) Representative images of lungs from untreated and PLX3397-treated lungs before tissue clearing (compare to **a** and **b**). Scale bar, 5 mm. (**e**) Representative images of treated and untreated tumours illustrate differences in TAM invasion. Scale bar, 1,000 μm. (**f**) NP fluorescence intensity corresponding to linear regions of interest placed across treated and untreated tumours, generating TAM intensity profiles. Tumours were normalized by size and plotted as a function of NP signal. (**g**) TAM localization was significantly altered within individual nodules. PLX3397-treated tumours have a greater ratio of NP signal in the tumour core relative to the rim (*n*=14 for each group, *P*=.025, 40–60% across tumour/0–10%+90–100%; unpaired *t*-test).

Figure 4 summarizes the results of CSF-1R treatment. The lungs of mice treated with PLX3397 had a much lower tumour burden than lungs in the control group (Fig. 4d): the pulmonary tumour volume in the treatment group was ∼6 mm^3^, whereas it was ∼93 mm^3^ in the control group (Fig. 4a, *n*=3 per group, *P*=0.015; unpaired *t*-test). Correlating tumour volume with TAM density for all tumour nodules (Fig. 4b) revealed that PLX3397 treatment significantly reduced individual tumour size (*n*=423, *P*<0.0001; unpaired *t*-test); however the average TAM density among tumours remained unchanged from the 16,500 TAM mm^−3^ observed in the untreated cohort (Fig. 4b,c, Supplementary Fig. 2b). Nonetheless, further spatial analyses by imaging individual tumour cross-sections (Fig. 4e) revealed that TAM in PLX3397-treated animals showed a distinct spatial distribution. In untreated mice, TAM density was highest in the tumour periphery as shown in cross-sectional TAM density profiles (Fig. 4f) averaged from of images through the centre of the tumour. In treated animals, TAM were instead more abundant at the centre of the tumour nodules (Fig. 4g, *n*=28, *P*=0.025; unpaired *t*-test), likely reflecting a response to tumour cell death and repair. These data indicate that CSF-1R inhibitors can effectively control lung cancer progression without affecting TAM density in whole tumours, but can profoundly alter TAM spatial distribution within lesions and/or their polar phenotype^41^,^42^,^43^,^44^.

### Nanoparticle based drug delivery to tumours

**Figure 5:**
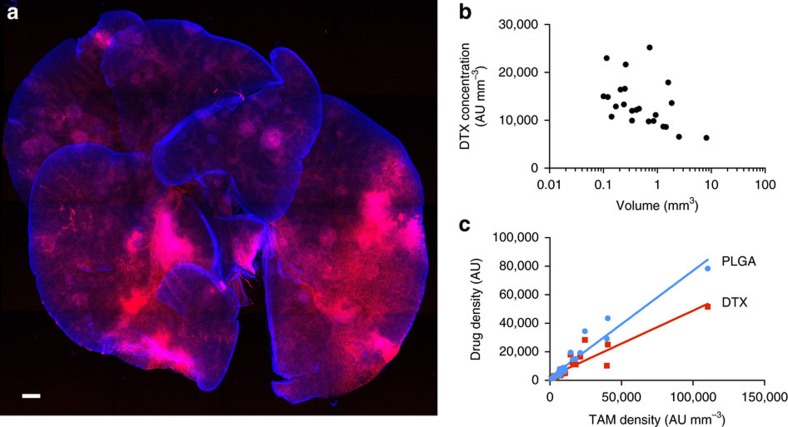
Nanoparticle-based taxane delivery to pulmonary tumours. A fluorescent docetaxel analogue (DTX; red) was incorporated into PLGA-PEG NPs. DTX was labelled with a silicon rhodamine NIR dye and the a BODIPY-TMR-labelled PLGA polymer was incorporated into the NP structure. The particle was injected in a KP tumour bearing mouse 35 days after tumour inoculation at a dose of 12 mg kg^−1^ DTX. (**a**) After clearing, DTX (red) is seen accumulating in DAPI-positive pulmonary tumour nodules. (**b**) Correlation between tumoural DTX concentration and metastases size. Note the drug heterogeneity. (**c**) Accumulation of either PLGA-PEG or DTX in tumours correlated with TAM density of lesions. Scale bar, 1,000 μm.

Prior research has shown that nanoencapsulated chemotherapeutics accumulate in cancers via the enhanced permeability and retention effect and can thus potentiate efficacy, while minimizing systemic toxicities^31^,^35^,^45^. Less clear, however, is how heterogeneous drug delivery manifests in metastatic contexts, and what governs such heterogeneity. To demonstrate the feasibility of imaging drug delivery with the clearing method, we administered dually labelled taxane-encapsulated polymeric nanoparticles to KP-bearing animals. Using a polymeric nano-formulation scheme previously optimized for intravital imaging^31^, near-infrared labelled docetaxel^46^ was encapsulated into a red-fluorescent polymeric micelle comprising the block co-polymer poly(lactic-*co*-glycolic acid)-*b*-polyethyleneglycol (PLGA-*b*-PEG) and the co-encapsulated fluorescent PLGA-BODIPY-TMR. Drug-loaded NPs were intravenously injected 18 h before killing. Figure 5 summarizes some of the results showing that taxane was present in each of the pulmonary metastases. Quite remarkably, certain metastases showed much higher accumulation, correlating well with TAM density (Fig. 5b,c). Furthermore, our data show that there was a tight correlation between PLGA-PEG and taxane delivery (R^2^= 0.94; Supplementary Fig. 6c). Previous efforts have demonstrated that TAM depletion decreases the efficacy of nanoparticle-based therapy, emphasizing the potential for TAM to serve as drug depots^31^.

## Discussion

To achieve optimal imaging from cleared tissue in orthotopic tumour sites, we experimented with several different methods of labelling cellular compartments: genetic reporters (different fluorescent proteins), intravenously injected intravital stains (lectins, TAM-targeting NPs, labelled antibodies) and post-clearing staining (DAPI, SYTO13) (Table 1). Our data show that the fluorescence of GFP/RFP and intravenously administered agents is preserved in the clearing process, whereas post-clearing immunolabelling is possible but time consuming given the slow antibody penetration through visceral tissues. Of particular interest in the current studies was the use of intravenously administered imaging agents such as the TAM-targeting dextran NP, a fluorescent and crosslinked version of ferumoxytol^47^. When administered intravenously before killing, these agents show broader distribution (and clearance in case of target absence), minimizing time required for labelling. It is also important to note that ferumoxytol could be used to assess TAM in cancer patients. Indeed, ferumoxytol is non-immunogenic^48^,^49^, Food and Drug Administration approved and has recently been shown to have sustained and rapid (within minutes) uptake into TAM^35^. Cellular density and IFP has been demonstrated to have little effect on the penetration of Ferumoxytol-VT680XL through tissue to access TAM^35^. While the nanoparticle shows no spatial dependency on TAM localization, TAM tend to primarily reside near tumour vasculature and are often associated with pro-angiogenic signalling themselves^50^. Consequently it could be used for clinical TAM assessment using magnetic resonance or PET imaging^34^.

By applying the clearing method to whole lungs, we were able to quantify lung tumour burden and observe TAM characteristics, such as density and tumour infiltration. This enabled detailed visualization of TAM and revealed notable heterogeneity in TAM density among lung tumour nodules in a single mouse. We quantified changes in tumour burden and TAM density in a KP pulmonary tumour model, as well as a GFP-labelled KP model in response to CSF-1R blockade (Supplementary Fig. 4). We also observed that different cancer models exhibited different overall TAM infiltration. This technique allows a practical throughput (Table 2) and cost-efficient method to increase our understanding of the tumour immune microenvironment. In the context of the rapidly advancing field of immuno-oncology, this method could be used to quantify the heterogeneity of immune response and its relationship to cancer and therapy.

The described method could also be adapted to other tumour and organ models and be expanded to other fluorescent protein reporter cells and mice^51^,^52^, fluorescently labelled drugs^53^,^54^,^55^ and biologicals^56^,^57^. Visualization of orthotopic tumours in other organs is a compelling extension to this work. The main limitations compared with the relatively ‘translucent' brain, which has a higher proportion of lipids that are removed by optical clearing, are the extent of light-absorbing and -scattering components in non-central nervous system organs and large tumours. Adaptation of intravital imaging tools^52^,^58^,^59^ for measuring cell–cell interaction and tumour proliferation to volumetric imaging could further reveal the influence of the tumour microenvironment on CSF-1R blockade and TAM polarization.

Post-clearing labelling techniques are constantly advancing with antibodies^24^,^60^ or FISH^61^ in brain tissue. We feel that these technologies, when adapted to oncology, may have the potential to piece together complex tumour architecture, cell–cell interaction networks and therapy-induced changes.

## Methods

### Animal models and cell lines

All lung clearing experiments were conducted in C57BL/6 mice (*n*=21), B6.129 P-Cx3cr1^*GFP/+*^ (*n*=1) and NOD.CB17-Prkdc^scid^/MERTK^*GFP/+*^ (*n*=1). All protocols were approved by the institutional IACUC review board. Mice were 5–7-week-old females at the time of tumour implantation. The tumour models consisted of the mutant Kras/P53 models^29^,^30^ and the Lewis lung cancer models^37^. The KP1.9 cell line was generated from lung adenocarinoma in C57BL/6, Kras^LSL G12D/+^; p53^fl/fl^ mice following infection with an adenovirus expressing Cre recombinase by intratracheal administration. KP cells were maintained in Iscove's DMEM media supplemented with 10% fetal bovine serum and 5% penicillin/streptomycin and all cell lines were routinely tested for contamination using mouse antibody production testing (VRL Laboratories) and assaying for mycoplasma (VRL Laboratories; Lonza MycoAlert). For tumour implantation, cells were injected into unanaesthetized B6 mice via tail vein injection of a single bolus of 2.5 × 10^6^ cells in 100 μl PBS. Parental KP1.9 cells were also transduced *in vitro* with a lentiviral vector expressing dLNGFR and eGFP using a bidirectional promoter system^62^. After transduction, cells were cloned by limiting dilution and selected for GFP expression to ensure uniformity of GFP brightness. A GFP high-expressing clone was selected for all experiments and 10^5^ cells were injected into B6 mice. Animals were killed and lungs harvested for imaging between 12 and 40 days. LLC cell line was purchased from the ATCC (American Type Culture Collection, Manassas, VA, USA. For CSF-1Ri therapy, a subset of mice were chosen at random for PLX3397 treatment (Selleck, Houston, TX, USA), which was administered i.p. at 30 mg kg^−1^ daily for 7 days. No exclusion criteria were applied.

### Mouse lung extraction and clearing protocol

The TAM-targeting NP (Ferumoxytol-VT680XL) was injected by tail vein 18 h before being killed^35^. The 18 h time point was previously determined to be optimal for NP vascular clearance and TAM uptake^35^. Rhodamine lectin Griffonia (Bandeiraea) Simplicifolia Lectin I (Vector Labs) was injected 1 h prior. Mice were then anaesthetized with isofluorane. Optimized CUBIC (Table 1): a small incision was made in the left ventricle of the heart and the lungs were perfused with 5 ml PBS through the right ventricle at a rate of about 2 ml min^−1^. Five millilitre of 4% formaldehyde (FA) was then perfused through the right ventricle at the same rate. The lungs were then inflated with 4% FA (2 ml) through the trachea. The lungs were then removed, placed in a conical tube containing 15 ml FA, and allowed to fix for an additional 3 h at room temperature (rt), with occasional application of vacuum to degas the lungs. The lungs were transferred to a glass vial containing 10 ml CUBIC solution and rocked for 24–48 h at 37 °C. Lungs were then mounted in fresh CUBIC solution between glass slides. Cleared lungs were stored at 4 °C with little loss in GFP or VT680 signal or resolution over several weeks. Nuclear labelling was performed with 4′,6-diamidino-2-phenylindole, dihydrochloride (DAPI) (Life technologies) or SYTO13 (Thermo Scientific) and airways were labelled with streptavidin-Alexa Fluor 750 (Thermo Scientific).

### Clearing solution

CUBIC reagent 1 solution: Water (145 ml) was added to a flask containing quadrol (N,N,N′,N′-Tetrakis(2-Hydroxypropyl)ethylenediamine (100 g, Sigma Aldrich) followed by addition of Urea (100 g, Sigma Aldrich). The mixture was placed on a heater/stirrer and warmed to about 50 °C until the solution became homogeneous. After cooling to room temperature, Triton X-100 (56 ml, Sigma Aldrich) was then added. The solution was then degassed under sonication and then vacuum. Lung tumour images acquired in CUBIC reagent 1 solution were determined to be equivalent to those acquired in the sucrose/urea-based CUBIC reagent 2 solution.

### Imaging methods

Images were obtained on Olympus FV1000 and IV100 confocal microscopes. Wide-field images were obtained by stitching a 5 × 4 grid of image stacks acquired with a × 2 NA 0.95 objective and presented as maximum intensity projections. A × 10 NA 0.6 water objective provided cellular resolution images,1.44 mm^2^ *x*–*y* coverage, and was suitable for two-photon imaging. × 20 NA 0.95 or × 40 NA 1.15 water objectives were also used when higher resolution was desired. All images were acquired in sequential mode and acquisition of stacks was started at maximum depth to offset bleaching effects. A 1 mm^3^ tumour could be imaged at cellular resolution (× 10 objective, 8 μs px^−1^ 512 × 512 px) in ∼10 min. Wide-field grids were acquired from both sides of the lungs in about 13 min per side. Grids were assembled in Image J, when necessary, mediastinum was digitally removed for presentation (Supplementary Fig. 5), and sides were co-registered for 3D viewing in FEI Amira (Supplementary Movie 3).

### Sample chamber

Tissue was mounted within a rubber gasket of appropriate thickness (1.5″ diameter, McMaster Carr 1182N128) between two microscopy grade slides (65 × 48 mm, thickness no. 1, Thermo Fisher Scientific 12-518-210), filled with clearing media, and sealed with silicone sealant (Dow Corning, McMaster Carr 75825A5).

### Post processing and analysis

3D renderings and movies were obtained using Bitplane Imaris and FEI Amira software. Two-dimensional images and co-localization plots were generated in Imagej. Cell counting in two-dimensional in cleared tissue and histological sections was performed with CellProfiler and in 3D with Bitplane Imaris. GraphPad Prism was used for graph generation and statistical analysis. Box plots are illustrated such that the box represents the 25th–75th percentile and whiskers represent the full range. Groups of tumours and animals were compared with an unpaired t-test.

### Flow cytometry

As described previously^35^, subcutaneous tumours with KP1.9 cells were harvested from C57BL/6 flanks 3 weeks after implantation, minced, and shaken at 600 r.p.m. with 0.2 mg ml^−1^ collagenase type I (Worthington Biochemical Corporation) in RPMI-1640 for 30 min at 37 °C. Digested samples were filtered (70 μm BD Falcon strainer); washed in PBS with 0.5% BSA and 2 mM EDTA; incubated with Fc-block (TruStain fcX anti-mouse CD16/32; clone 93; Biolegend) for 15 min at 4 °C; and labelled with antibodies as indicated for 45 min at 4 °C. Flow cytometry (LSRII, BD Biosciences) labelled tumour cells (CD45^−^ EpCAM^+^), TAM (CD45^+^ CD11b^+^ Ly6C^-^ Lin^-^ CD11c^+^ F4/80^+^), lymphocyte-like cells (CD45^+^ CD11b^-^ Lin^+^), along with CD45- EpCAM- host-cell populations. Antibodies included EpCAM (clone G8.8; eBioscience); CD45 (clone 30-F11; Biolegend), F4/80 (clone BM8; Biolegend), CD11c (clone N418; Biolegend), Ly6C (clone HK1.4; Biolegend); and CD11b (clone M1/70; BD Biosciences). The lineage (Lin) antibody mix contained anti-CD90.2 (clone 53–2.1), anti-B220 (clone RA3-6B2), anti-NK1.1 (clone PK136), anti-CD49b (clone DX5), anti-Ter119 (cloneTER-119) and anti-Ly6G (clone 1A8) (all BD Biosciences). 7-aminoactinomycin D (7-AAD, Sigma Aldrich) excluded dead cells. VT680 fluorescence was directly assessed using the LSRII flow cytometer, FlowJo v.8.8.7 (Tree Star, Inc.) and MATLAB.

### Immunohistochemistry

Tumour-bearing mice were injected with the fluorescent NP 18 h before killing. In the same manner as for clearing, mice were mice were anaesthetized with isofluorane and perfused with PBS followed by 4% FA through the right ventricle. Lungs were further fixed for 3 h, washed with PBS and placed in 30% sucrose in PBS overnight. The lung tissue was embedded in O.C.T. compound (Sakura Finetek) and snap-frozen in a 2-methlbutane bath on dry ice. Serial 20 μm thick sections were prepared and stained with CD68 (clone: FA-11, Bio-Rad, MCA1957A488, 1:25) and CD206 (clone: C068C2, BioLegend, 141704, 1:25) followed by biotinylated anti-rat IgG (1:100) and streptavidin-DyLight 594 (Vector Laboratories, Inc. 1:600). KP 1.9 and RAW 264.7 cells were seeded overnight at 20,000 cells per well in an eight-well glass slide (Millipore). Cells were fixed in 4% FA for 15 min at rt, fixative was quenched with glycine and cells were permeabilized with 0.5% Tween-20 for 5 min at rt. Cells were blocked for 30 min with Odyssey blocking buffer (Licor) and incubated with anti-CSF-1R-Alexa Fluor 594 antibody conjugate (BioLegend, Clone AFS98, 135520, 1:50) in Odyssey buffer for 1 h. Cells were washed with PBS for 1 h. To identify nuclei, the sections were counterstained with 4′,6-diamidino-2-phenylindole, dihydrochloride (DAPI) (Life technologies, 1:3000). For analysis, the images were captured by using a fluorescence microscope (Olympus BX63) equipped with Neo sCMOS Monochrome Camera (ANDOR).

### Nanoparticle synthesis and administration

Using a formulation previously described^31^, a silicon-rhodamine conjugated docetaxel analogue^46^, PLGA-PEG (HOOC-PEG(5.5 kDa)-b-PLGA(75/25)(8.3 kDa), Advanced Polymer Materials) and PLGA(50:50 PL:GA, 30–60 kDa, Sigma Aldrich)-BODIPY-TMR were dissolved in 2 ml acetone and rapidly added to a stirring beaker containing 2 ml water. After evaporation of acetone, particles were filtered through a 0.45 μm membrane filter and concentrated on a 50 kDa molecular weight cutoff filter. NP size and drug loading were performed as described previously^31^. NP size was determined by DLS (Malvern Zetasizer) to be 80 nm, PDI 0.29. Drug loading was 87% for a final concentration of 2.35 mM as determined by ultraviolet absorption (NanoDrop). 100 μl NP solution amounting to a 12 mg kg^−1^ dose of DTX was injected intravenously. After 18 h, the animal was killed and prepared for clearing.

### Data avaibility

The data that support the findings of this study are available from the corresponding author upon reasonable request.

## Additional information

**How to cite this article:** Cuccarese, M. F. *et al*. Heterogeneity of macrophage infiltration and therapeutic response in lung carcinoma revealed by 3D organ imaging. *Nat. Commun.* **8,** 14293 doi: 10.1038/ncomms14293 (2017).

**Publisher's note**: Springer Nature remains neutral with regard to jurisdictional claims in published maps and institutional affiliations.

## Supplementary Material

Supplementary Information Supplementary Figures

Supplementary Movie 1 Volume rendering of 1 mm3 tumor, acquired at 10x.

Supplementary Movie 2 Volume rendering of tumor and airway, acquired at 20x.

Supplementary Movie 3 Volume rendering of a whole tumor-bearing lung.

## Figures and Tables

**Table 1:** Effect of clearing protocol on affinity ligands.

**Protocol**	**Endogenous label**	**IV-label**	**Stains**	**IHC**	**Time**	**Transparency**
Optimized CUBIC	Preserved, GFP, RFP, collagen (SHG)	Preserved, rhodamine-lectin	Yes, DAPI, SYTO13, Streptavidin Alexa-Fluor 750	Possible but slow	3 days	Good
CUBIC	Preserved	Preserved	Yes	Possible But Slow	5 days	Good
ScaleS	Preserved	Preserved	Yes	Possible but slow	5 days	Moderate

**Table 2:** Overview of times required for different experiments steps.

**Process**	**Average time per mouse**	**Comments**
Intravital administration of affinity ligands	10 min	Two iv injections: TAM marker 18 h before killing, lectin 1 h prior.
*In vivo* perfusion and pulmonary resection	20 min	Perfusion through right ventricle with PBS followed by 4% FA ensures lung perfusion and fixation. Whole-animal perfusion also possible through left ventricle.
Post-perfusion fixation	3 h	Room temperature. Apply vacuum 1–2 × to remove air from alveoli.
Clearing	24–48 h	Performed under gentle rocking, 37 °C. Lungs prone to trapping air if shaking too rapid.
Mesocopic imaging of entire lung	20 min	Since lungs are of similar size, process is streamlined by using the same imaging grid program over multiple samples.
× 10 Confocal imaging of ∼1 mm tumour	20 min	
× 10 Imaging of entire lung	Hours	Results in extremely large file sizes. Spot imaging of tumours is a practical alternative to whole organ imaging at × 10.
