# Supplementary material for: Heterogeneity of macrophage infiltration and therapeutic response in lung carcinoma revealed by 3D organ imaging
Source: Nat Commun. 2017 Feb 8;8:14293. doi: 10.1038/ncomms14293 (PMC5309815; doi:10.1038/ncomms14293)

Supplementary Figure 1. Optical tissue clearing procedure, example images. **a**. Process of tissue clearing. Mice are perfused through the right ventricle (left ventricle clipped, red arrow) with PBS followed by 4% formaldehyde and further fixed in 4% FA for 3 h. Lungs are mounted between two large cover slips and imaged on a confocal microscope. **b**. Images at the center of a tumor as viewed from above and side. Scale bars 100 µm. **c**. Identification of non-fluorescent tumors with the nuclear dye SYTO13. Scale bar 1000 µm. **d**. Example of healthy lung tissue stained with vasculature and TAM markers. Scale bar 1000 µm, 100 µm respectively. **e**. Labeling of endogenous bronchial biotin with a fluorescent streptavidin conjugate (Movie 2). Scale bar 500 µm. **f**. Use of 2-photon second harmonic generation to image intratumoral collagen. Scale bar 100 µm.

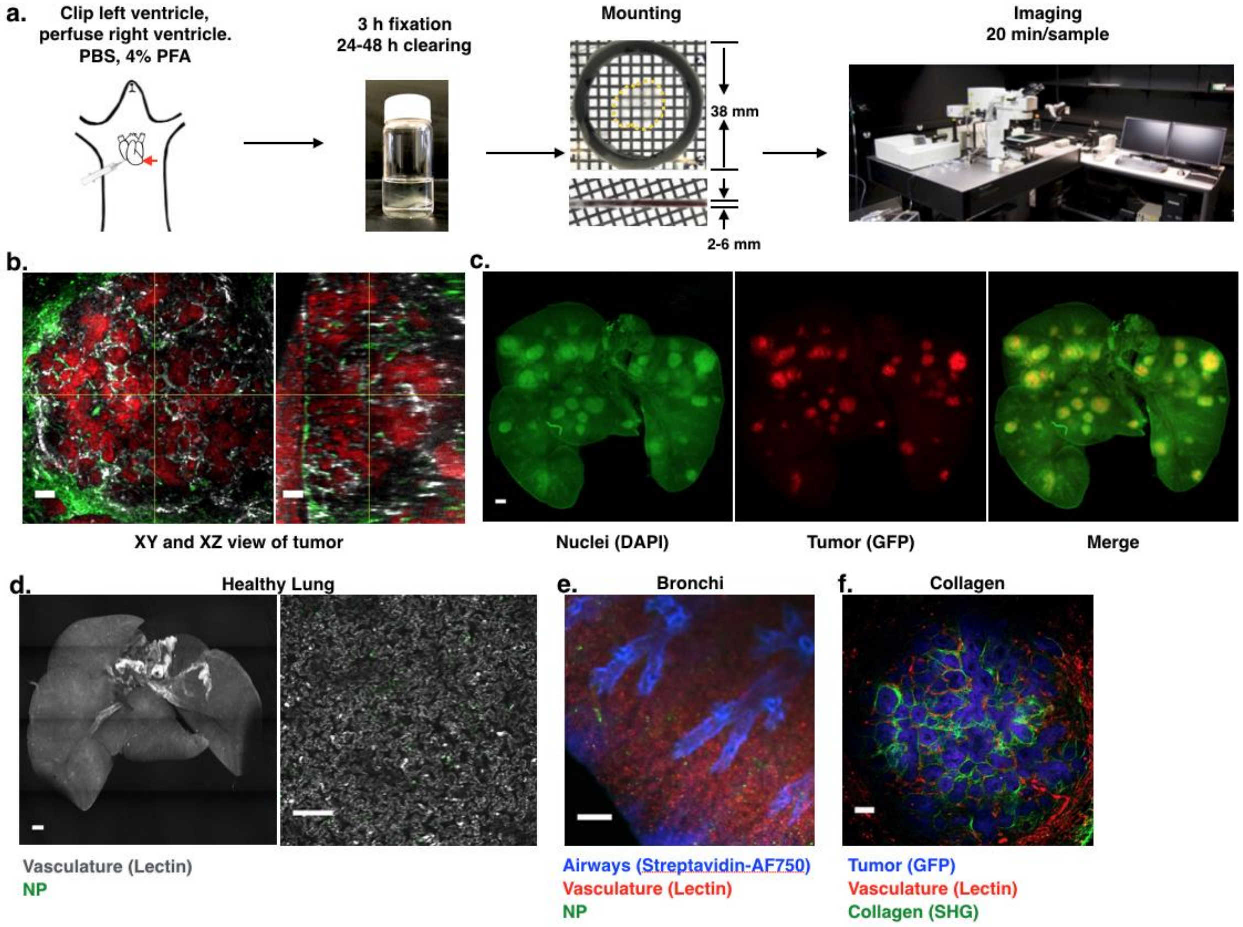

Supplementary Figure 2. Confirmation of nanoparticle uptake in TAM by colocalization with MERTK-GFP reporter mouse. Human HT1080 fibrosarcoma cells expressing mApple red fluorescent protein were injected either subcutaneously (a) or in a dorsal skinfold window chamber (b) in NOD-SCID MERTK-GFP mice. NP was then injected and imaged after 18 h following the optimized tissue clearing protocol (a) or directly in the window chamber (b). Pixel colocalization was performed in imagej (a) and cell segmentation was performed in CellProfiler. Scale bars 100 µm.

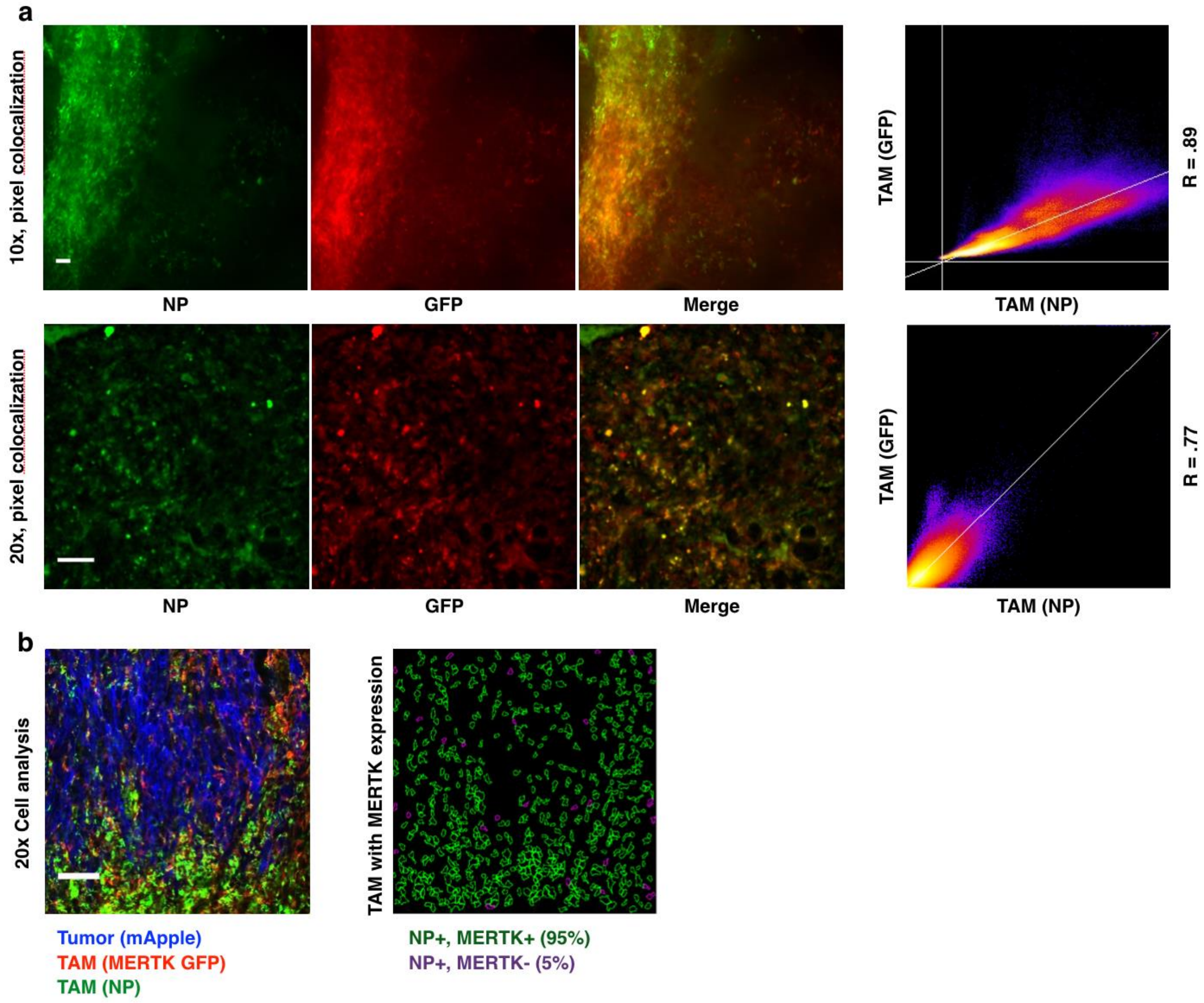

Supplementary Figure 3. Cell counting method. TAMs were counted by first relating wide-field integrated fluorescence density (normalized to background autofluorescence) to high resolution images of TAMs. Tumors were selected from wide-field images and images were acquired at the center of each tumor at high resolution. TAMs were counted for 20 tumors with CellProfiler. Cell counts were then related to fluorescence density in wide field to generate a fluorescence-density to TAM-density conversion factor. To evaluate predictive value, cell numbers inferred from wide field images were compared to the computationally calculated values, revealing a correlation coefficient of .9042, p<.0001; paired t-test. The fluorescence density to cell number ratio was applied to new wide-field images to rapidly acquire TAM density in wide field images.

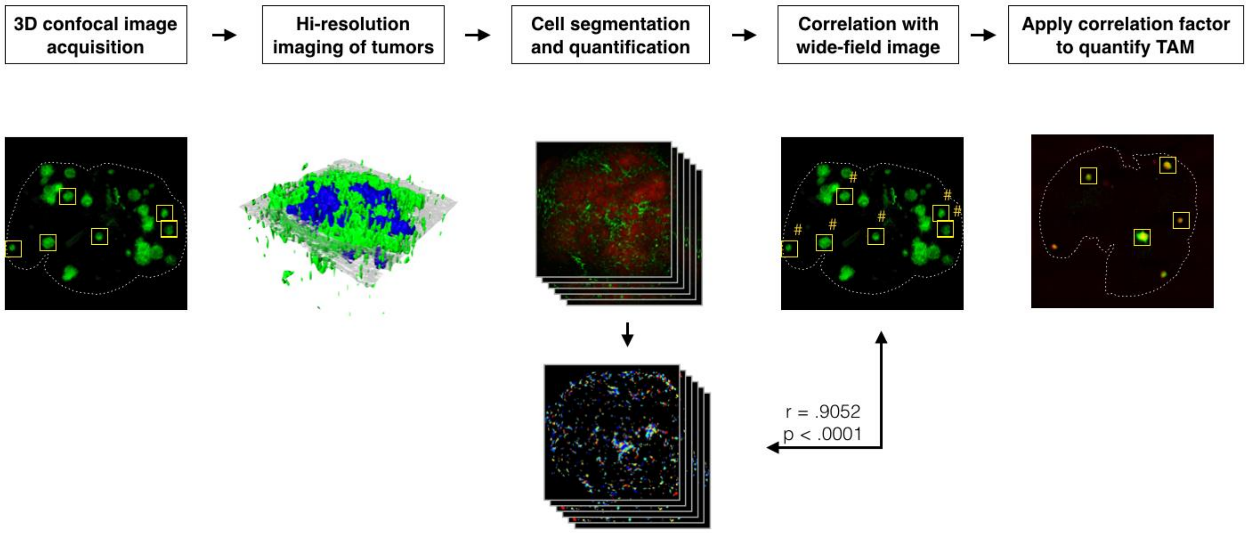

Supplementary Figure 4. Tumor response in KP-GFP lung tumors treated with PLX3397. **a**. Two Cohorts of mice were inoculated with a GFP-labeled KP cell line and treated with PLX3397. In cohort 1, lungs were resected just before and after 7 days of PLX3397 treatment. In cohort 2, all mice were sacrificed after 7 days of treatment. **b**. Comparison of TAM density in KP and KP-GFP tumors. The GFP labeled tumors recruited significantly more TAMs than the unlabeled cell types. TAM density was not significantly affected by CSF-1R blockade in either model.

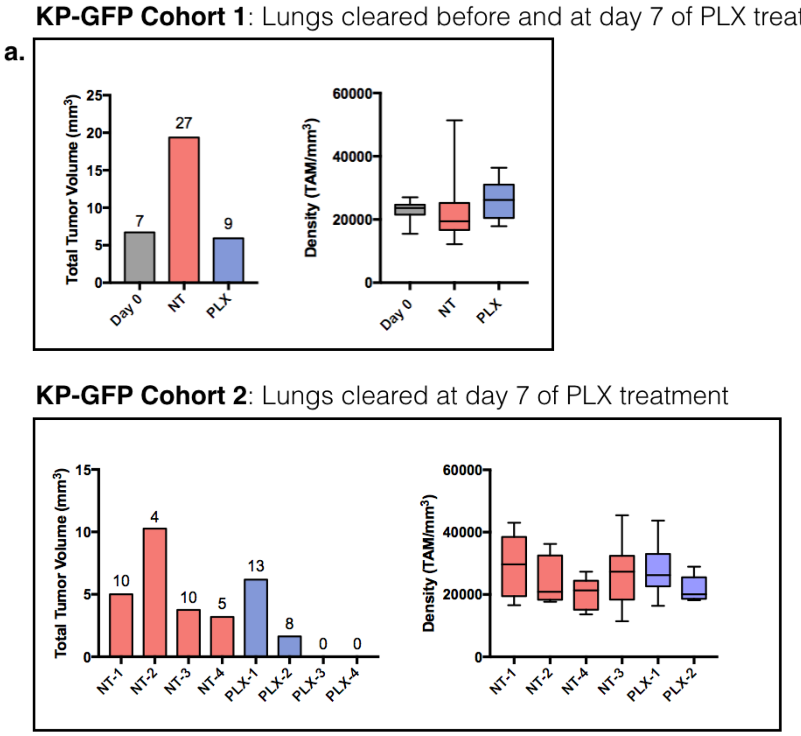

b.

ns (KP-NT vs KP-PLX); ns (KP-GFP-NT vs KP-GFP-PLX)

	KP-NT	KP-PLX
KP-GFP-NT	P<.0001	P<.0001
KP-GFP-PLX	P<.0001	P<.0001

Supplementary Figure 5. Wide-field TAM images of lungs from CSF-1Ri cohort. Wide-field images of TAM in KP tumor bearing mice with and without 7 days of PLX3397 treatment. Tumors were delineated with TAM and vasculature channels and analyzed for TAM density presented in Fig. 4. Mediastinum was digitally removed for clarity. Scale bar 1000 µm.

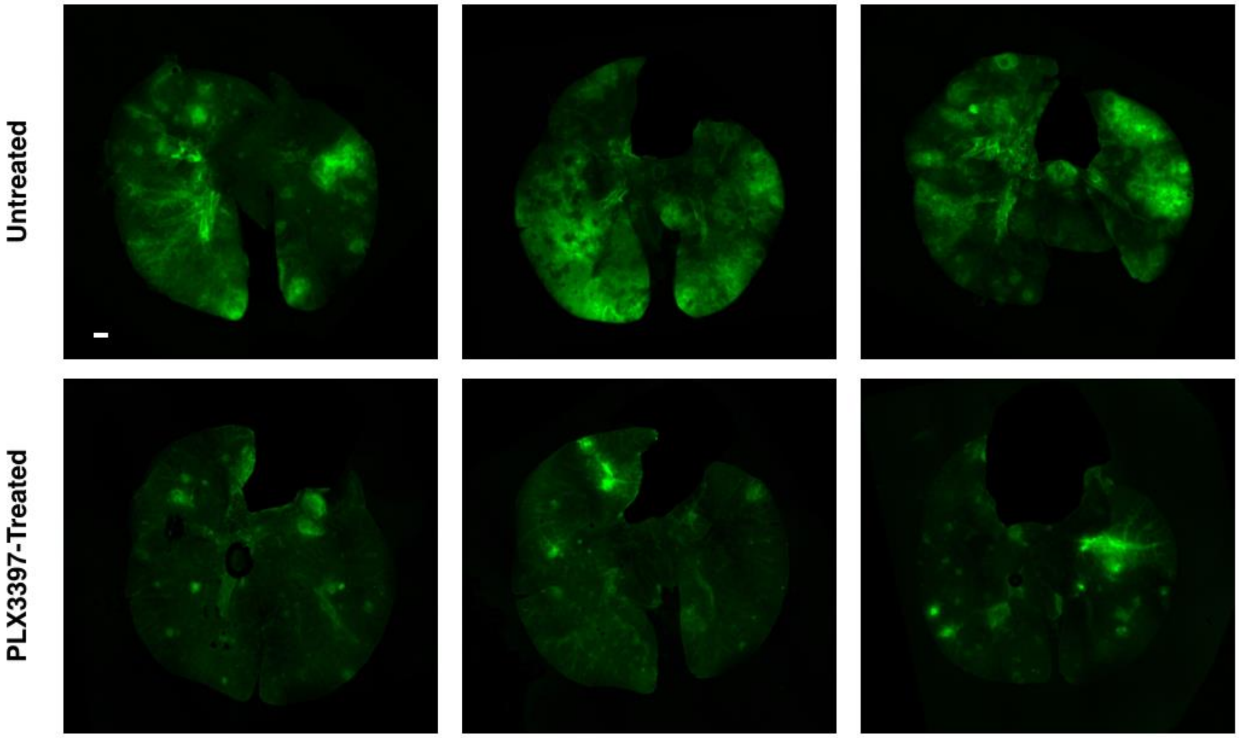

Supplementary Figure 6. Nanoparticle based taxane delivery. a. Wide field image of CX3CR1, PLGA and DTX. Strong correlation is observed between PLGA and DTX, indicating drug association with the polymer nanoparticle. Scale bar 1000 µm. **b**. High-resolution analysis of PLGA-PEG nanoparticle (PLGA), DTX and iron oxide nanoparticle (NP) in CX3CR1-GFP positive cells. Scale bar 10 µm. **c**. Correlation between DTX and PLGA density in tumors. **d**. Correlation between DTX and TAM density in tumors.

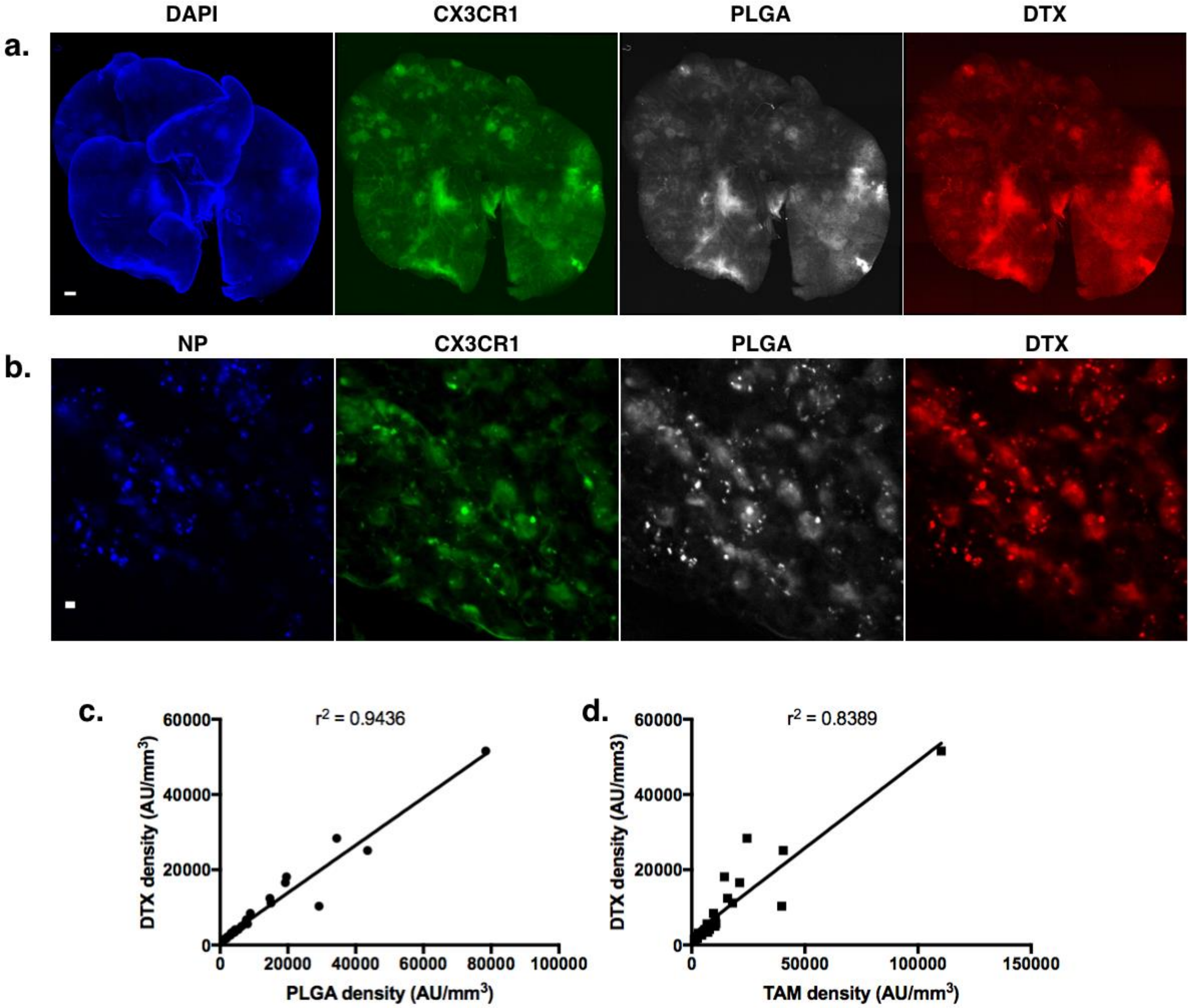

Supplementary Figure 7. Comparison of images acquired in CUBIC-1 and CUBIC-2 solutions; Comparison of tissue in unrestricted and 2mm chambers. b. KP-GFP tumors as imaged in CUBIC-1 and CUBIC-2 solutions at depths of 100 and 600 µm. Scale bar 50 µm.

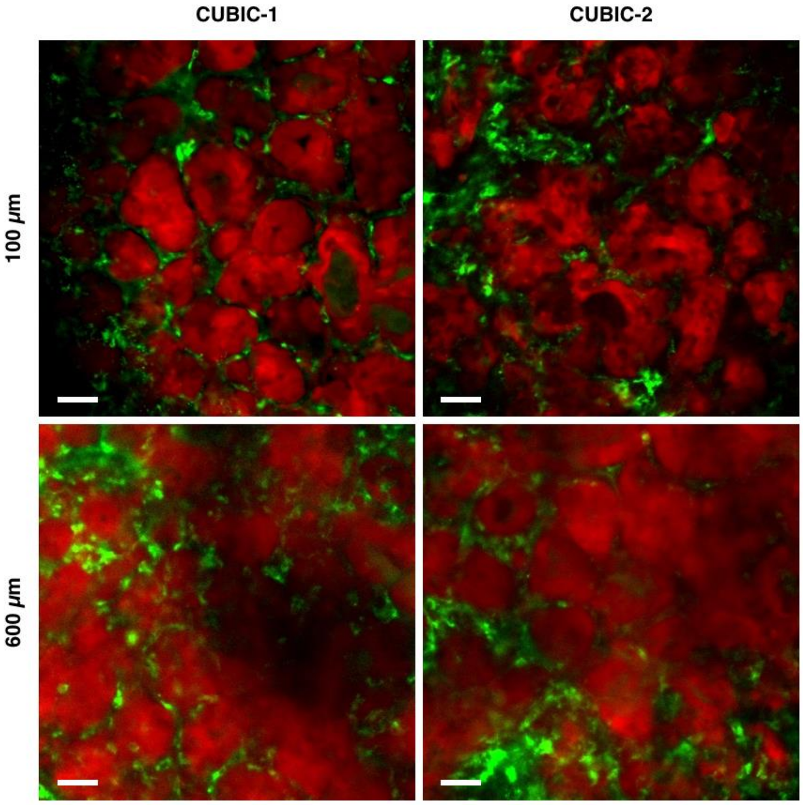

Supplementary Figure 8. Expression of CSF-1R on KP 1.9 cells and sensitivity to PLX3397. a. PLX3397 is not toxic to KP 1.9 cancer cell line or RAW 264.7 murine macrophages up to 100 µM. b. Absence of CSF-1R on KP cells. RAW 264.1 cells are known to express the receptor.

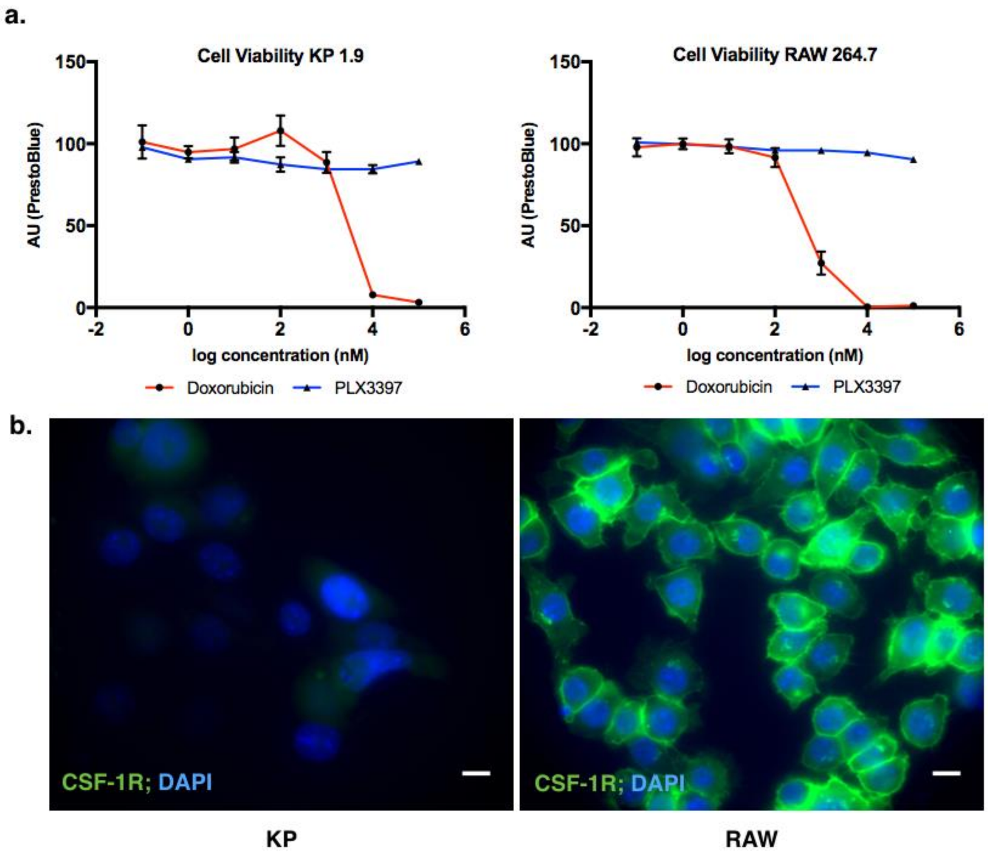

Supplementary Figure 9. Acquisition and co-registration of a small tumor in unrestricted chamber and a 2 mm chamber. a. Vasculature was stained with rhodamine lectin and image stacks were acquired for the same tumor in the two chambers. Scale bars 100 µm (10x), 50 µm (20x). b. The images were co-registered in 3D without applying distortion or scaling. c. Illustration of co-registration accuracy. Scale bar 100 µm. Displacement vectors (black arrows) were calculated between vascular surfaces in the tumor within the two chambers and plotted as a function of magnitude (d.) (FEI Amira). 92.8 % of 1.3×10^6 vectors were < 10 µm.

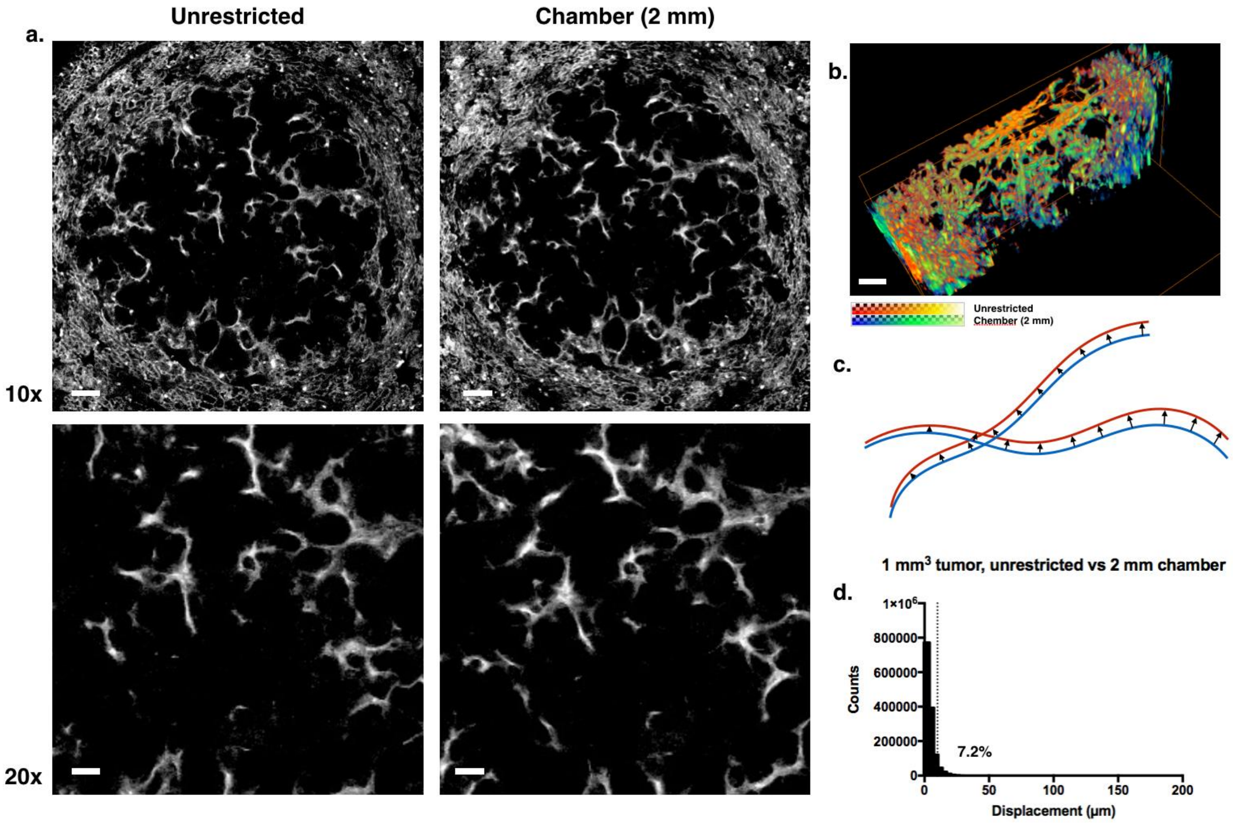

Supplementary Figure 10. Effect of clearing on lung transparency. Images from 8 tumor bearing lungs before and after clearing. Note that clearing yields nearly transparent lungs in each example facilitating whole lung microscopic imaging. For experimental detail see the sample examples in Fig. 4d.

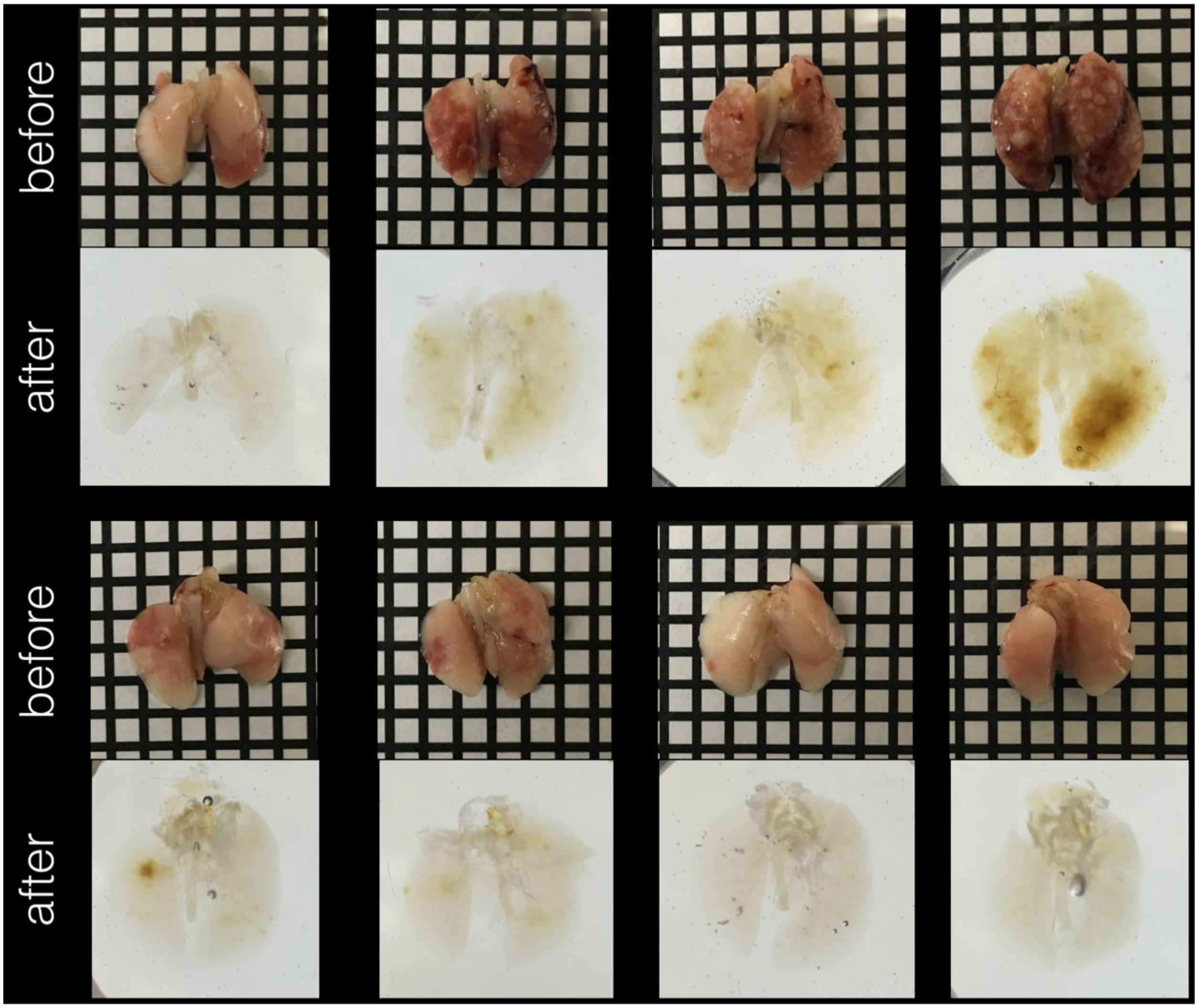

Supplement: Supplementary Information — Supplementary Figures [file ncomms14293-s1.pdf]
